# Osteocyte Shape and Mechanical Loading

**DOI:** 10.1007/s11914-015-0256-1

**Published:** 2015-02-07

**Authors:** René F. M. van Oers, Hong Wang, Rommel G. Bacabac

**Affiliations:** 1 Department of Oral Cell Biology, ACTA - University of Amsterdam and VU University Amsterdam, MOVE Research Institute Amsterdam, Gustav Mahlerlaan 3004, 1081 LA Amsterdam, The Netherlands; 2 Department of Dental Materials Science, ACTA - University of Amsterdam and VU University Amsterdam, MOVE Research Institute Amsterdam, Amsterdam, The Netherlands; 3 Medical Biophysics Group, Department of Physics, University of San Carlos, Talamban Campus, Cebu City, Philippines

**Keywords:** Osteocyte shape, Mechanical loading, Collagen fiber orientation, Mechanosensation

## Abstract

There is considerable variation in the shape of osteocyte lacunae, which is likely to influence the function of osteocytes as the professional mechanosensors of bone. In this review, we first discussed how mechanical loading could affect the shape of osteocyte lacunae. Recent studies show that osteocyte lacunae are aligned to collagen. Since collagen fiber orientation is affected by loading mode, this alignment may help to understand how mechanical loading shapes the osteocyte lacuna. Secondly, we discussed how the shape of osteocytes could influence their mechanosensation. In vitro, round osteocytes are more mechanosensitive than flat osteocytes. Altered lacunar morphology has been associated with bone pathology. It is important to know whether osteocyte shape is part of the etiology.

## Introduction

The adult skeleton is continuously remodeled by bone-resorbing osteoclasts and bone-forming osteoblasts. This process is believed to be regulated by osteocytes, former osteoblasts that have been buried in the bone matrix. They remain in contact with the cells on the bone surface via cytoplasmic processes that are connected via gap junctions. The matrix immediately around the osteocyte cell body and processes does not mineralize, thus forming the network of lacunae and canaliculi. Their strategic location in this lacuna-canalicular network enables osteocytes to fulfill their role as mechanosensors [1–4]. The osteocyte cell bodies are not free-floating in the lacunae but anchored via their cell processes that radiate in all directions [5, 6]. The cell processes in turn are believed to be anchored to the canalicular walls via tethering proteins [7, 8].

How osteocytes sense mechanical loading of the bone is still a subject of ongoing research. Bone strains are much lower (<0.3 %) than the direct cell strains needed (1–3 %) to provoke a metabolic response from osteocytes in vitro [3]. Nicolella et al. [4, 9] suggest that microstructural strains within the bone, near lacunae and microcracks, are much higher than macrostructural strains and thus able to stimulate osteocytes directly. An older theory was that bone cells sense electric potentials caused by deformation of bone under load [10], but this theory has fallen out of favor since this piezoelectric effect is exhibited by dry bone and barely by wet bone. It was subsequently suggested that osteocytes sense streaming electric potentials caused by the movement of ions with loading-induced fluid flow through the canaliculi [11]. From there, it was a small step to suggest that the fluid flow itself was the main stimulus [1, 3, 12, 13]. Theoretical models predicted that fluid flow in the narrow space between osteocyte cell process and surrounding canaliculus can generate shear stresses on the cell membrane of 0.8–3 Pa during physiological loading [3, 12]. In vitro, pulsating fluid flow with a mean stress of only 0.5 Pa and 5-Hz pulses of ±0.02 Pa provoked a doubled release of nitric oxide (NO) and a fivefold release of prostaglandins E_2_ and I_2_ compared to static culture [14, 15]. The fluid flow sensation may further be amplified by hoop stress on the osteocyte cell process [16], by drag forces on tethering fibers between cell process and canalicular wall [7, 17] or by bumps in the canalicular wall [18].

There is considerable variation in the shape of osteocyte lacunae between different bone types and regions. Let us consider an osteocyte lacuna as an ellipsoid with principal axes of length *a*, *b*, *c*. There are four possible ellipsoid shapes: scalene (*a* > *b* > c), prolate (*a* > *b* = *c*), oblate (*a* = *b* > *c*), spherical (a = b = c). Everyday examples of scalene, prolate, and oblate ellipsoids are almonds, rugby balls, and Smarties, respectively. All four types have been used to describe osteocyte lacunae. Osteocyte lacunae in woven bone have an irregular spherical shape [19, 20], while lacunae in lamellar bone typically have a scalene ellipsoid (almond-like) shape [19, 21–23]. Lacunae in mouse calvariae were found to be more oblate [24]. All these shape variations are likely to have an effect on the lacunar deformation and subsequent osteocyte mechanosensation. The topic of this review is the interaction between mechanical loading and osteocyte shape. This interaction is twofold: firstly, how mechanical loading affects the shape of osteocyte lacunae; secondly, how the shape of osteocytes influences their mechanosensation.

## From Mechanical Loading to Osteocyte Shape

Several researchers have hinted at a link between osteocyte shape and bone loading patterns. Vatsa et al. [24] found a difference in osteocyte morphology between mouse fibulae and calvariae, the fibular osteocytes being more elongated and the calvarial osteocytes more spherical or oblate. They suggested that these differences in morphology reflected differences in mechanical loading, since loading is more unidirectional in long bones and more bidirectional in calvariae. But how osteocytes adjust their shape to the habitual loading of their bone region was left unanswered.

Here, it is important to know whether the shape of the osteocyte lacuna is determined when the former osteoblast is buried and the surrounding matrix mineralizes or whether the osteocyte can modify the shape of its lacuna at a later stage. Several studies have indicated that osteocytes have the ability to mobilize perilacunar calcium (osteocytic osteolysis) during periods of calcium depletion, as well as the ability to synthesize new matrix upon calcium repletion [25–27]. However, although these processes can increase or reduce the size of the lacuna, it is questionable whether they can actively modify its shape and orientation. This would require that the osteocyte localizes the secretion of osteolytic enzymes or new matrix proteins at a particular side of its cell body.

Marotti [21] convincingly demonstrated a correlation between lacunar orientation and collagen fiber orientation in human osteonal bone. He found that the major axis of lacunae is parallel to the collagen in both longitudinally structured osteons (collagen parallel to osteon axis) and transversely structured osteons (collagen perpendicular to osteon axis). While this early work received little attention, a recent study by Kerschnitzki et al. [28••] again demonstrated this correlation. A link between lacunar and collagen orientation suggests that lacunar shape is determined during the formation phase. Indeed, osteoblasts in periosteum are aligned with the collagen they produce [29]. This raises the question whether the osteoblasts direct the collagen or the collagen directs the osteoblasts. Matsugaki et al. [30•] cultured primary osteoblasts on fabricated collagen substrates and found that the osteoblasts aligned to the collagen substrate. It also appeared that newly synthesized matrix was oriented along the cells, indicating an interactive relation between collagen and osteoblast orientation.

**Fig. 1:**
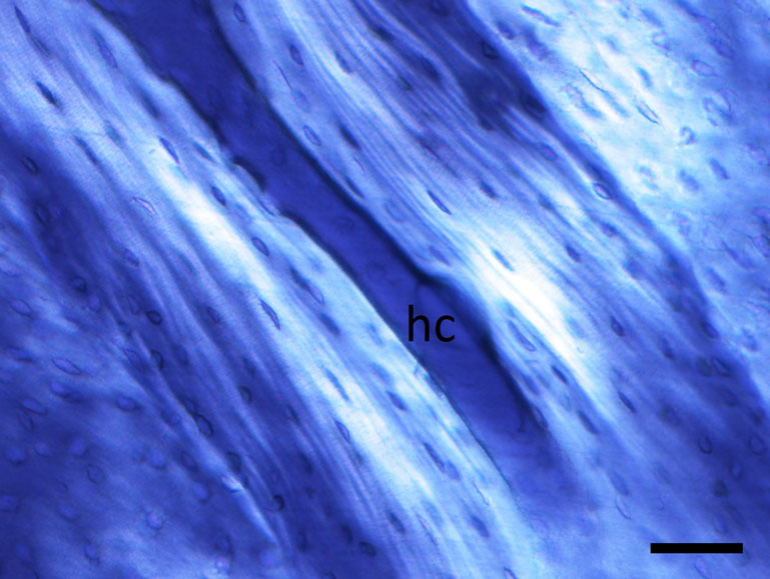
Polarized light microscopy of an osteon in a longitudinal section of cow femur. *Bright areas* indicate that collagen runs parallel to the plane of section. Note that osteocyte lacunae appear longer in the bright areas near the Haversian canal (*hc*) compared to the dark outer areas of the osteon. *Scale bar* = 50 μm

The link with collagen orientation provides a way to understand how mechanical loading affects osteocyte shape. Bone consists primarily of organic collagen and inorganic hydroxyapatite. It is the collagen that gives the bone its tensile strength, like the steel wires in reinforced concrete. It makes the bone resilient to fracture [31] and is crucial for the connection of ligaments and tendons to the bone [32]. Bromage et al. [33] compared transverse sections of different bones with polarized light microscopy. Bone sections appear bright in polarized light microscopy when collagen fibers are parallel to the section plane and dark when they are perpendicular to the section plane (Fig. 1). Bones loaded primarily in tension, such as the gibbon ulna, appeared dark, suggesting that collagen runs longitudinal (perpendicular to a transverse section = along the long axis of the bone). Bones that experience more compression appeared much brighter, indicating that collagen runs more transverse to the long axis of the bone. The association of longitudinal collagen with tension and transverse collagen with compression has also been observed in sheep heel bones, which experience tension on one side and compression on the other side [34, 35•].

**Fig. 2:**
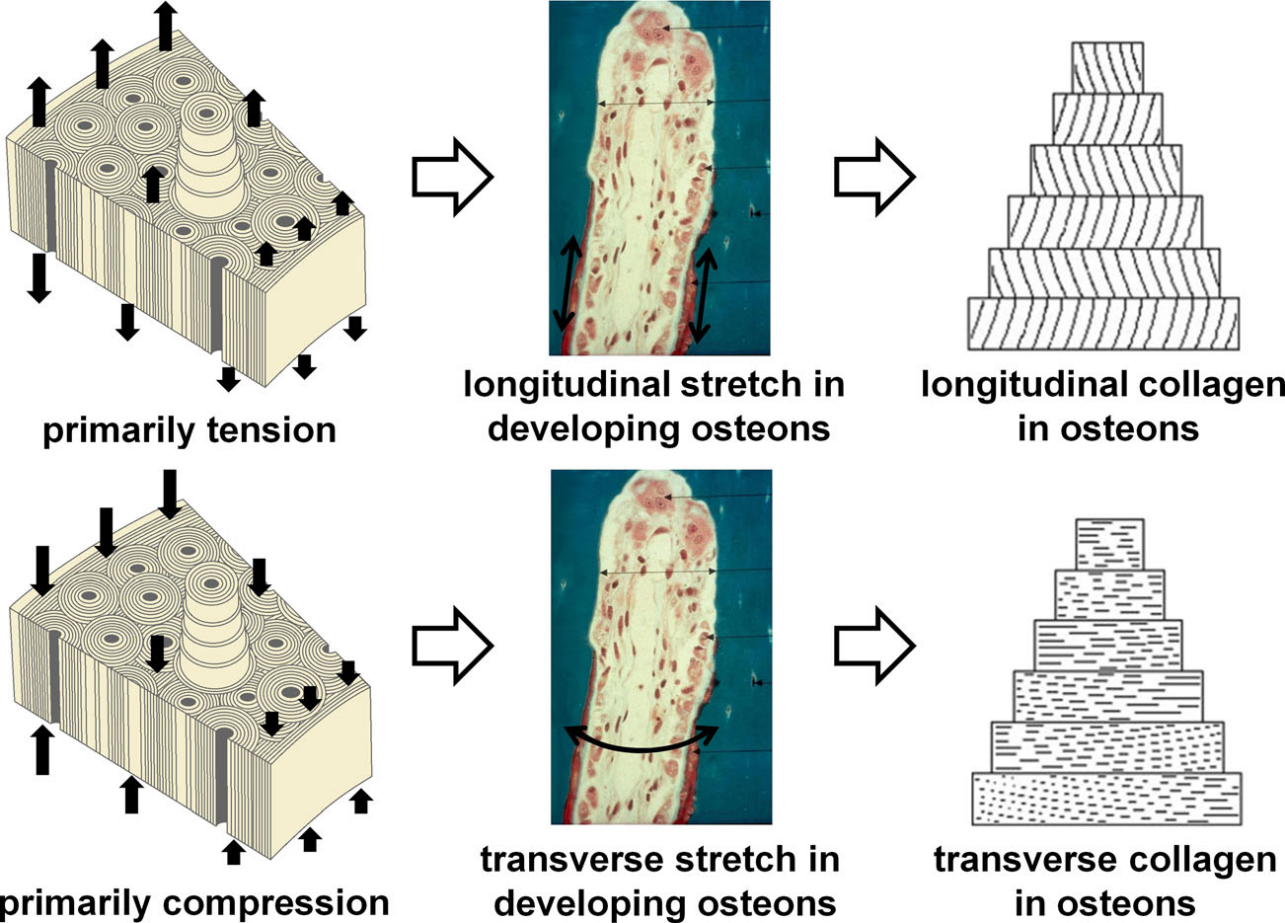
Hypothetical explanation of observed differences in preferential collagen fiber orientation with loading mode (tension or compression)

How could the dominant loading mode, tension or compression, produce bone with longitudinal or transverse collagen? Osteons align to the dominant loading, whether that is tension or compression [36]. We previously proposed a mechanobiological explanation for this alignment [37–40]: strain concentrations at the lateral sides of the osteonal tunnel induce osteocyte signals to repel the digging osteoclasts, orienting them in the loading direction. This mechanism does not discriminate between tension and compression: osteoclasts would avoid both compressed and tensed bone. Though both compression and tension guide the osteonal tunnel in the longitudinal direction [36], they differ in the orientation of stretch (positive strain) on the tunnel wall: cavities in bone under compression dilate transversely, while cavities under tension are stretched longitudinally (Fig. 2). The orientation of stretch in the osteoid layer thus coincides with the preferential orientation of collagen in the finished osteon. Osteocyte shape, in turn, might also reflect the stretch in the osteoid layer of its formation.

Reality, however, is more complicated. While tension or compression may drive a preferential orientation of collagen via longitudinally or transversely structured osteons, it is harder to explain the so-called alternately structured osteons in which collagen orientation changes from lamella to lamella. Marotti et al. [19] found that osteocyte lacunae in these osteons are mainly located in longitudinally structured lamellae, with their major axis at an angle of 26°–27° from the osteon axis. Apart from tension and compression, shear is a major strain mode experienced by osteons. Skedros et al. [35•] suggested that shear could play a significant role in the development of osteon morphotypes.

The idea that collagen aligns to stretch is already being explored in soft tissue research [41, 42]. It is also known from soft tissue research that collagen can orient to external stretch in the absence of cells, since unstrained collagen fibers are more prone to degradation [43]. However, the strains that direct collagen orientation in soft tissue research are much larger than strains in the bone. On the other hand, stretch in the osteoid layer would be caused not only by external loading but also by osteoblast cell traction [44]. Hence, the osteoblasts may be crucial in orienting the collagen to stretch from external loads.

## From Osteocyte Shape to Mechanosensation

Differences in lacunar shape can affect the mechanical signal that osteocytes feel. Differently shaped or oriented lacunae are likely to have a different volumetric deformation under a specific load. Changes in volumetric deformation will change the load-induced fluid flow, which osteocytes are believed to feel [1, 3, 12, 13]. Given the abovementioned hypothetical effects of loading mode (tension or compression) on osteocyte shape, it would be interesting to investigate how differently shaped and oriented lacunae deform under different loading modes. Lacunar shape may also affect the generation of microdamage, which is known to trigger osteocyte apoptosis [45] and subsequent bone remodeling [46]. In a two-dimensional finite element study, Prendergast and Huiskes [47] found that lacunae perpendicular to a tensile load were more affected by microdamage than those parallel to the load.

Differently shaped osteocyte cell bodies may have a different mechanosensitivity to the same mechanical signal. This has been explored in in vitro studies, where cells of different shape, outside their lacunar space, can be subjected to the same mechanical stimulus such as fluid flow or substrate strain. Bacabac et al. [48] compared the elastic properties and mechanosensitivity of round (partially adherent or suspended) and flat (adherent) MLO-Y4 osteocytes, using optical tweezers. They found that round osteocytes had stiffness well below 1 kPa, whereas flat osteocytes had stiffness above 1 kPa. The found stiffness value of round cells was recently corroborated by analysis of osteocyte deformation under fluid flow [49]. Round osteocytes were more mechanosensitive than flat osteocytes [48]. Whereas round osteocytes required a 5-pN deforming force in order to release NO, flat osteocytes needed a thousand-fold higher force indentation.

The underlying cytoskeletal structure in flat cells supports the formation of stress fibers and focal adhesion centers, which are not expected in suspended cells. The functional differences between round and flat osteocytes therefore suggest that the architecture of the cytoskeleton is a prerequisite to mechanosensation. Note further that the influence of the lacunar shape would impact the transfer of stress information to the cell. It is possible that bone cell mechanosensation is dependent on both the cellular and lacunar shape.

Altered osteocyte mechanosensitivity has been suggested as a potential cause for bone loss. Van Hove et al. [50] compared osteocyte morphology in human proximal tibial bone with relatively low (osteopenic), medium (osteoarthritic), and high (osteopetrotic) BMD. Osteopenic osteocytes were relatively large and round (8.9 × 15.6 × 13.4 μm), compared to osteoarthritic (8.4 × 17.3 × 12.2 μm) and osteopetrotic (5.5 × 11.1 × 10.8 μm) osteocytes. On the other hand, McCreadie et al. [51] found no significant difference in lacunar size or shape between women with and without osteoporotic fracture, although there was a large range of sizes and shapes in both groups. Even with a significant correlation between osteocyte shape and osteoporosis, one should be cautious about inferring causation from correlation. Altered lacunar shape might be a cause of osteoporosis via altered mechanosensitivity or it might be an effect of osteoporosis, e.g., due to increased matrix strains in an osteoporotic bone structure subjected to physiological loads, or it might share a common cause with osteoporosis, e.g., if the hormonal changes that alter osteoclastic/blastic activity also induce osteocytic osteolysis.

Geometric (or structural) considerations are important in generating a qualitative insight on how a single osteocyte would feel stress when bone is loaded. However, it has also been shown in vitro that the rate of loading correlates with the accumulated molecular response of bone cells to fluid shear stress [52, 53]. This provides further insight on how noise plays a role in differentiated responses to fluid shear stress by bone cells. Thus, osteocyte sensitivity to stress is not solely dependent on how it might deform under the influence of stress. Rather, the dynamic nature of the stimulating stress plays a vital role in how osteocytes would choose to respond.

## Conclusions

There is considerable variation in the shape of osteocyte lacunae, which is likely to influence the function of osteocytes as the professional mechanosensors of bone. In this review, we discussed firstly how mechanical loading could affect the shape of osteocyte lacunae and secondly how the shape of osteocytes could influence their mechanosensation.

Renewed interest in the link between lacunar and collagen orientation may help to understand how mechanical loading shapes the osteocyte lacuna. Longitudinal collagen has been associated with tensile loading, and more transverse collagen with compressive loading. This preferential collagen fiber orientation would coincide with the orientation of stretch (positive strain) in the osteoid layer of developing osteons under these loading modes. Osteocyte shape, following collagen fiber orientation, might thus reflect the stretch in the osteoid layer of its formation.

Variations in osteocyte shape between bone regions with different loading modes raise the question whether this represents an adaptive response. However, if osteoblasts align (with) the collagen and become entombed with this shape, then the main load adaptation was in the collagen, and the osteocyte shape could be just a secondary effect. Furthermore, if we speculate about the benefits of particular osteocyte shapes in particular bone regions, we first have to ask ourselves the following question: should the osteocyte be more sensitive to the habitual loading that guided its shape or to changes in loading that may warrant an adaptive response? Here, it might not suffice to consider a rather static picture of how stress falls on osteocytes. The interplay between short and long duration of loading bouts may have independent or correlated effects on cellular responses to stress.

Variation in lacunar and osteocyte cell shape undoubtedly has an effect on osteocytic mechanosensation and subsequent control of bone remodeling. Firstly, variation in lacunar shape will alter the direct cell strain, fluid flow, and microdamage inputs to the osteocyte. Furthermore, the shape of the osteocyte cell body affects its sensitivity to these inputs. Changes in osteocyte morphology have been associated with osteoporosis. It is important to know whether these changes represented a cause or an effect of the osteoporosis.
